# Supplementary material for: The Systems Biology Research Tool: evolvable open-source software
Source: BMC Syst Biol. 2008 Jun 29;2:55. doi: 10.1186/1752-0509-2-55 (PMC2446383; doi:10.1186/1752-0509-2-55)
Supplement: Additional file 1 — SBRT Archive. An archive of the current version of the Systems Biology Research Tool. [file 1752-0509-2-55-S1.zip › sbrt-1.4.0/doc/developers_guide/api/sbrt/shell/text/FormatException.html]

FormatException


|  |  |  |
| --- | --- | --- |
| **Overview** \| **Package** \| **Class** \| **Use** \| **Tree** \| **Deprecated** \| **Index** \| **Help** | |  |
| **PREV CLASS**   **NEXT CLASS** | **FRAMES**    **NO FRAMES**     **All Classes** |  |
| SUMMARY: NESTED \| FIELD \| CONSTR \| METHOD | DETAIL: FIELD \| CONSTR \| METHOD |  |


---


## sbrt.shell.text Class FormatException

```
java.lang.Object
  java.lang.Throwable
      java.lang.Exception
          java.lang.RuntimeException
              sbrt.shell.ApplicationException
                  sbrt.shell.text.FormatException
```

**All Implemented Interfaces:**: java.io.Serializable

---

``` public class FormatException extends ApplicationException ```

This exception type is used to indicate a formatting error
has ocurred. If a string is unparsable because it does
not conform to the expected format, this exception should
be thrown.

**Author:**
:   This class was written and documented by
    Jeremiah Wright while in the Wagner lab.

**See Also:**: Serialized Form

---

**Constructor Summary**

`FormatException(java.lang.String message)`
  
          Constructs a new format exception with
the provided error message.

`FormatException(java.lang.String message,
java.lang.Throwable cause)`
  
          Constructs a new format exception with
the provided error message and cause.


**Method Summary**

**Methods inherited from class sbrt.shell.ApplicationException**

`getMessage`

**Methods inherited from class java.lang.Throwable**

`fillInStackTrace, getCause, getLocalizedMessage, getStackTrace, initCause, printStackTrace, printStackTrace, printStackTrace, setStackTrace, toString`

**Methods inherited from class java.lang.Object**

`clone, equals, finalize, getClass, hashCode, notify, notifyAll, wait, wait, wait`

**Constructor Detail**

### FormatException

```
public FormatException(java.lang.String message)
```

:   Constructs a new format exception with
    the provided error message.

    **Parameters:**: `message` - a complete, informative, well-formed sentence describing the error that ocurred (which is saved for later retrieval by the `Throwable.getMessage()` method).

---


### FormatException

```
public FormatException(java.lang.String message,
                       java.lang.Throwable cause)
```

:   Constructs a new format exception with
    the provided error message and cause.

    **Parameters:**: `message` - a complete, informative, well-formed sentence describing the error that ocurred (which is saved for later retrieval by the `Throwable.getMessage()` method).: `cause` - the cause (which is saved for later retrieval by the `Throwable.getCause()` method). (A null value is permitted, and indicates that the cause is nonexistent or unknown.)


---


|  |  |  |
| --- | --- | --- |
| **Overview** \| **Package** \| **Class** \| **Use** \| **Tree** \| **Deprecated** \| **Index** \| **Help** | |  |
| **PREV CLASS**   **NEXT CLASS** | **FRAMES**    **NO FRAMES**     **All Classes** |  |
| SUMMARY: NESTED \| FIELD \| CONSTR \| METHOD | DETAIL: FIELD \| CONSTR \| METHOD |  |


---
